# Cigarette smoke induces PTX3 expression in pulmonary veins of mice in an IL-1 dependent manner

**DOI:** 10.1186/1465-9921-11-134

**Published:** 2010-10-04

**Authors:** Nele S Pauwels, Ken R Bracke, Tania Maes, Geert R Van Pottelberge, Cecilia Garlanda, Alberto Mantovani, Guy F Joos, Guy G Brusselle

**Affiliations:** 1 Laboratory for Translational Research in Obstructive Pulmonary Diseases, Department of Respiratory Medicine, Ghent University Hospital, Ghent, Belgium; 2 Laboratory for Immunology and Inflammation, Istituto Clinico Humanitas IRCCS, Rozzano, Milan, Italy; 3 Department of Translational Medicine, University of Milan, Milan, Italy

## Abstract

**Background:**

Chronic obstructive pulmonary disease (COPD) is associated with abnormal inflammatory responses and structural alterations of the airways, lung parenchyma and pulmonary vasculature. Since Pentraxin-3 (PTX3) is a tuner of inflammatory responses and is produced by endothelial and inflammatory cells upon stimuli such as interleukin-1β (IL-1β), we hypothesized that PTX3 is involved in COPD pathogenesis.

**Methods and Results:**

We evaluated whether cigarette smoke (CS) triggers pulmonary and systemic PTX3 expression *in vivo *in a murine model of COPD. Using immunohistochemical (IHC) staining, we observed PTX3 expression in endothelial cells of lung venules and veins but not in lung arteries, airways and parenchyma. Moreover, ELISA on lung homogenates and semi-quantitative scoring of IHC-stained sections revealed a significant upregulation of PTX3 upon subacute and chronic CS exposure. Interestingly, PTX3 expression was not enhanced upon subacute CS exposure in IL-1RI KO mice, suggesting that the IL-1 pathway is implicated in CS-induced expression of vascular PTX3. Serum PTX3 levels increased rapidly but transiently after acute CS exposure.

To elucidate the functional role of PTX3 in CS-induced responses, we examined pulmonary inflammation, protease/antiprotease balance, emphysema and body weight changes in WT and Ptx3 KO mice. CS-induced pulmonary inflammation, peribronchial lymphoid aggregates, increase in MMP-12/TIMP-1 mRNA ratio, emphysema and failure to gain weight were not significantly different in Ptx3 KO mice compared to WT mice. In addition, Ptx3 deficiency did not affect the CS-induced alterations in the pulmonary (mRNA and protein) expression of VEGF-A and FGF-2, which are crucial regulators of angiogenesis.

**Conclusions:**

CS increases pulmonary PTX3 expression in an IL-1 dependent manner. However, our results suggest that either PTX3 is not critical in CS-induced pulmonary inflammation, emphysema and body weight changes, or that its role can be fulfilled by other mediators with overlapping activities.

## Background

Chronic obstructive pulmonary disease (COPD), a primarily cigarette smoke (CS)-induced disease, is a major cause of chronic morbidity and mortality worldwide [1,2]. COPD is characterized by progressive and largely irreversible airflow limitation caused by obstructive bronchiolitis and emphysema which are associated with an abnormal inflammatory response of the lungs to noxious particles or gases [2]. Several mechanisms are involved in the disease pathogenesis: inflammatory cell recruitment to the lungs, imbalance between proteolytic and anti-proteolytic activity, oxidative stress and apoptosis/proliferation imbalance [3]. Besides major abnormalities in the airways, changes in pulmonary vessels represent an important component of COPD pathology [4]. Moreover, some patients with COPD exhibit low-grade systemic inflammation that is often associated with extrapulmonary (systemic) effects, such as weight loss and cardiovascular disease [5-7]. However, the precise molecular mechanisms whereby CS triggers abnormal pulmonary and systemic manifestations remain unclear.

Pentraxins are a superfamily of soluble pattern recognition receptors characterized by a cyclic multimeric structure [8]. Pentraxin 3 (PTX3), the prototypic long pentraxin which is highly conserved between mice and humans, differs from short pentraxins (C-reactive protein [CRP] and serum amyloid P [SAP]) in many aspects such as cellular source, regulation of the production and function [8]. PTX3 is, in contrast with the hepatically derived short pentraxins, mainly produced by inflammatory cells [9] and endothelial cells [10,11], which allows it to act locally at sites of infection and inflammation. It is produced in response to the pro-inflammatory cytokines IL-1β and tumor necrosis factor-α (TNF-α) and microbial components such as LPS, a component of gram-negative bacteria, which is also present in CS [10-12].

PTX3 has major roles in innate immunity and inflammation [8]. It interacts with specific pathogens such as Klebsiella pneumoniae [13], and apoptotic cells [14], thereby contributing to their clearance. Patients with COPD are frequently colonized with bacteria in the lower airways [15] and several reports described cell death of structural cells such as epithelial cells [16] and endothelial cells [17] in human emphysema. Moreover, PTX3 mediates angiogenesis by influencing Fibroblast Growth Factor-2 (FGF-2) activity [18] and is a marker of endothelial dysfunction reflecting vascular inflammatory state in many diseases such as small vessel vasculitis [19]. Importantly, exposure to environmental CS induces pulmonary angiogenesis in mice [20]. PTX3 is also described as an early indicator of acute myocardial infarction (AMI) in humans [21] and its cardioprotective role, by modulating the reperfusion-associated inflammatory response and tissue damage, is dependent on the IL-1RI pathway, as determined in a mouse model of AMI [22]. PTX3 appears to be a stronger predictor of cardiovascular mortality than C-reactive protein (CRP), a short pentraxin elevated in patients with COPD [23]. Therefore, we put forward the hypothesis that PTX3 plays a critical role in COPD, a chronic inflammatory disease where multiple inflammatory and resident cells are involved and multiple organs are affected.

In this study, we evaluated the impact of CS on pulmonary PTX3 expression in wild-type (WT) mice. We next investigated PTX3 expression in lungs of mice deficient for the IL-1 pathway (IL-1RI KO mice) to elucidate the molecular mechanism. We also examined the *in vivo *functional role of PTX3, by measuring pulmonary inflammation including lymphoid aggregate formation and emphysema in WT and Ptx3 KO mice upon CS exposure. We next examined if CS triggers systemic PTX3 expression and if PTX3 deficiency affects CS-induced systemic manifestations such as body weight changes.

## Methods

### Animals

Homozygous breeding pairs of C57BL/6J WT mice and IL-1RI knockout (KO) mice (B6.129S7-*Il1r1^tm1Imx^*) were obtained from The Jackson Laboratory (Bar Harbor, ME, USA) and bred in the animal facility at Faculty of Medicine and Health Sciences, Ghent University (Ghent, Belgium). Additionally, mice with a heterozygous targeted mutation in the Ptx3 gene, backcrossed 11 generations onto the C57BL/6 background, were obtained from A. Mantovani (Istituto Clinico Humanitas IRCCS, Rozzano, Milan, Italy) [13]. Heterozygous breeding pairs were bred in the animal facility and the offspring was genotyped using a protocol described by C. Garlanda et al. [13]. Male homozygous Ptx3 KO mice and homozygous wild-type (WT) littermates were used for the described experiments. Animals of 7-13 weeks were divided into age-and body weight-matched groups and maintained in standard conditions under a 12 h light-dark cycle, provided a standard diet (Pavan, Brussels, Belgium) and chlorinated tap water *ad libitum*. All *in vivo *manipulations were approved by the local Ethics Committee for animal experimentation of the Faculty of Medicine and Health Sciences, Ghent University.

### Cigarette smoke (CS) exposure

Groups of 10 mice were exposed to CS, as described previously [24]. Briefly, the animals received mainstream CS of 5 reference cigarettes (2R4F without filter; University of Kentucky, Lexington, KY, USA) 4 times a day with 30-min smoke-free intervals. An optimal smoke/air ratio of 1/6 was obtained. The mice were exposed for 3 days (acute), 4 weeks (subacute) or 24 weeks (chronic). The control groups were exposed to room air. The body weight of the mice were measured at the beginning and the end of the chronic experiment. Immediately after smoke exposure, carboxyhaemoglobin (COHb) fractions in blood were measured in CS-exposed (8.35 ± 0.47%) and air-exposed mice (0.65 ± 0.25%) (n = 4).

### Collection of serum

At 1 h, 6 h or 24 h after the last exposure, mice were sacrificed by an i.p. injection of pentobarbital (CEVA-Sanofi, Paris, France). Subsequently, blood was sampled from the orbital sinus and processed for the collection of serum.

### Bronchoalveolar lavage (BAL)

Bronchoalveolar lavage was performed as previously described [25]. Briefly, lungs were first lavaged using 3 times 300 μl HBSS, free of Ca^2+ ^and Mg^2+ ^and supplemented with 1% BSA, followed by 3 times 1 ml HBSS supplemented with 0.6 mM EDTA, via a tracheal cannula. The six lavage fractions were pooled, centrifuged, and the cell pellet was finally resuspended in 200 μl buffer (PBS supplemented with 1% BSA, 5 mM EDTA and 0.1% sodium azide). Subsequently, total cell counts were obtained using a Bürker chamber and differential cell counts (on at least 400 cells) were performed on cytocentrifuged preparations after May-Grünwald (Sigma-Aldrich, St. Louis, MO) and Giemsa staining (VWR, West Chester, PA, USA). Discrimination of neutrophils was obtained based on standard morphologic criteria. Flow cytometric analysis of BAL cells was performed to enumerate macrophages, dendritic cells (DCs) and CD4^+ ^and CD8^+ ^T-lymphocytes.

### Lung harvest and preparation of lung single-cell suspensions

Following BAL, the pulmonary and systemic circulation was rinsed with saline, supplemented with 5 mM EDTA. The left lung was excised for histology, as previously described [26]. The right lung was used for the preparation of lung homogenate (middle lobe) and single-cell suspension (major lobe), as described previously [26]. Briefly, the lung was thoroughly minced, digested, subjected to red blood cell lyses, passed through a 50 μm cell strainer and kept on ice until labelling. Cell counting occurred with a Z2 particle counter (Beckman-Coulter Inc., Fullerton, CA, USA).

### Labelling of BAL cells and lung single-cell suspension for flow cytometry

The cells were first incubated with FcR blocking antibody (anti-CD16/CD32, clone 2.4G2) to reduce nonspecific binding. Secondly, the labelling reactions were performed to discriminate DCs, macrophages and T-lymphocytes. All reactions were performed on ice using monoclonal Abs obtained from BD Pharmingen (San Diego, CA, USA). The DCs and macrophages were discriminated using the methodology described by Vermaelen and Pauwels [27]. Briefly, DCs were characterized as CD11c-bright (APC-conjugated anti-CD11c; HL3), low autofluorescent and MHC class II-high (PE-conjugated anti-I-A[b]; AF6-120.1) population. Macrophages are identified as the CD11c-bright and high autofluorescent cell population. Mouse T cell subpopulations in lung single cell-suspensions were identified by the following antibodies (Abs): FITC-conjugated anti-CD4 (GK1.5), FITC-conjugated anti-CD8 (53-6.7), APC-conjugated anti-CD3 (145-2C11) and PE-conjugated anti-CD69 (H1.2F3), a marker for activation of T-lymphocytes. Finally, all samples were incubated with 7-Amino-actinomycin D for exclusion of dead cells (BD Pharmingen, San Diego, CA, USA).

Flow cytometry data acquisition was performed on a dual-laser FACS Calibur™flow cytometer running CellQuest™software (BD Biosciences, San Diego, CA, USA). FlowJo Software (Tree Star Inc., Ashland, OR, USA) was used for data analysis.

### Preparation of lung tissue homogenate

The middle lobe of the right lung was snap-frozen (in liquid nitrogen) and stored at -80°C until further analysis. The lobes were transferred to tubes containing 1 ml T-PER Tissue Protein Extraction Reagent containing Halt™Protease Inhibitor Cocktail Kit (Thermo Fisher Scientific, Waltham, MA, USA) and homogenized on ice using TissueRuptor (Qiagen, Hilden, Germany). The homogenates were centrifuged (10000 × g for 5 min at 4°C) and the middle layer was transferred to microcentrifuge tubes. Total protein concentration was measured using the Bradford Protein Assay (Bio-Rad Laboratories, Hercules, CA, USA). Lung tissue homogenates were diluted with T-PER containing Cocktail Kit to a final protein concentration of 500 μg/ml.

### PTX3, VEGF-A and FGF-2 ELISA

We determined PTX3 levels (BAL fluid, lung homogenate and serum), VEGF-A (lung homogenate) and FGF-2 levels (lung homogenate) using commercially available ELISA kits with a sensitivity of 12 pg/ml, 3 pg/ml and 8 pg/ml respectively (R&D systems, Minneapolis, MN, USA). ELISA was performed following the manufacturer's instructions.

### Histology of the lung

The left lung was fixed by gentle infusion of fixative (4% paraformaldehyde) through the tracheal cannula [24]. After excision, the lung was immersed in a fresh fixative for 2 h. The lung lobe was embedded in paraffin and cut into 3 μm transverse sections, followed by immunohistochemical and chemical staining. Photomicrographs were captured using KS400 image analyze platform (Zeiss, Oberkochen, Germany) and analyzed quantitatively.

### Immunohistochemistry and quantification of pentraxin-3

To evaluate pentraxin-3 expression in lung tissue, sections were subjected to PTX3 staining using anti-PTX3 antibody. First, tissue sections were incubated with Boehringer blocking agent with 0.3% triton and primary antibody anti-PTX3 (Alexis Biochemicals, Farmingdale, NY) or isotype rabbit Ig (Abcam, Cambridge, UK). Subsequently, the slides were incubated with PowerVision poly-horse radish peroxidase (HRP)-anti-rabbit (Immunovision Technologies, Burlingame, CA, USA) and stained with 3,3'-diaminobenzidine ([DAB] Dako, Glostrup, Denmark). Sections were counterstained with hematoxylin and mounted using mounting medium (Thermo Fisher Scientific). Photomicrographs were taken of all PTX3 positive veins and the area of the endothelial cells was marked manually on the digital representation of the vein using KS400 image analyze platform (Zeiss). The surface area covered by the stain was measured by the software (KS400) and its value was normalized to the length of internal perimeter (P) of the vein.

### Quantification of peribronchial lymphoid aggregates

To evaluate the presence of lymphoid aggregates in lung tissue after chronic smoke exposure, lung sections were subjected to double staining for CD3 (T-lymphocyte marker) and B220/CD45R (B-lymphocyte marker), as described previously [28]. Briefly, the sections were first incubated with Boehringer blocking agent with 0.3% triton and primary antibody anti-CD3 (Dako) or isotype rabbit IgG1,κ (Abcam). Subsequently, the slides were incubated with PowerVision poly-HRP-anti-rabbit (Immunovision Technologies) and stained with DAB (Dako).

Secondly, the sections were stained with anti-B220/CD45R biotin (RA3-6B2) or isotype IgG2a,κ biotin after Boehringer blocking with 0.3% triton. Both antibodies for the second step were purchased from BD Pharmingen. Subsequently, the slides were incubated with rat-on-mouse alkaline phosphatase (AP)-polymer kit (Biocare Medical, Concord, CA, USA) and stained with Vector Blue (Vector Laboratories, Burlingame, CA, USA). Thirdly, sections were counterstained with 0.5% methylgreen (Sigma-Aldrich) and mounted using Vectamount mounting medium (Vector Laboratories).

Lymphoid aggregates, defined as dense accumulations of at least 50 cells, were counted in the tissue area surrounding the airways (airway perimeter 0-2000 μm). Results were expressed as counts relative to the numbers of airways per lung section.

### Emphysema measurement

Emphysema, a structural disorder of the lung parenchyma characterized by airspace enlargement, is quantified after 24 weeks of air or CS exposure by measurement of the mean linear intercept (Lm), as described in detail previously [24,29,30], using image analysis software (Image J1.36b).

### RNA preparation and real-time RT-PCR

RNA was extracted from lung tissue using the miRNeasy Mini kit (Qiagen), following manufacturer's instructions. Expression of VEGF-A, FGF-2, MMP-12 and TIMP-1 mRNA, relative to HPRT mRNA (Hypoxanthine guanine phosphoribosyl transferase, a reference gene) was determined by real-time RT-PCR using the TaqMan Gene Expression assays (Applied Biosystems, Foster City, CA, USA) including the respective primers/fluorogenic probe mix specific for each molecule. Real-time RT-PCR reactions were set up in triplicate using identical reverse transcription and amplification conditions for each of the molecules and the reference gene. Reverse transcription was performed at 48°C for 30 minutes. Amplification conditions consisted of: 10 minutes at 95 °C, 45 cycles of 10 s at 95 °C and 15 s at 60 °C. Reaction samples had a final volume of 20 μl consisting of Universal Master mix, RNase inhibitor, MuLV Rtase, the specific primer/probe mix (Applied Biosystems) and 10 ng total RNA. Amplifications were performed, using a LightCycler480 detection system (Roche, Basel, Switzerland).

### Statistical analysis

Statistical analysis was performed with Sigma Stat software (SPSS 15.0, Chicago, IL, USA) using non-parametric tests (Kruskall-Wallis; Mann-Whitney U). Reported values are expressed as mean ± SEM. P-values < 0.05 were considered to be significant.

## Results

### Cigarette smoke (CS) exposure increases expression of pentraxin-3 in pulmonary endothelial cells

**Figure 1:**
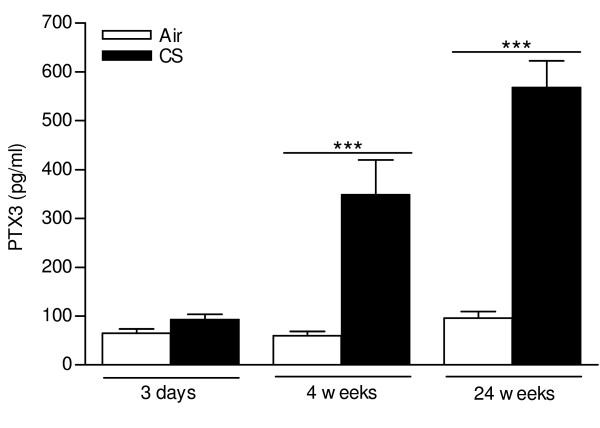
**PTX3 expression in lung homogenate by ELISA**. PTX3 protein levels in lung homogenate of WT mice in the acute (3 days), subacute (4 weeks) and chronic (24 weeks) experiment, as measured by ELISA. Data are expressed as mean ± SEM (N = 8-10 animals/group; *** p < 0.01).

**Figure 2:**
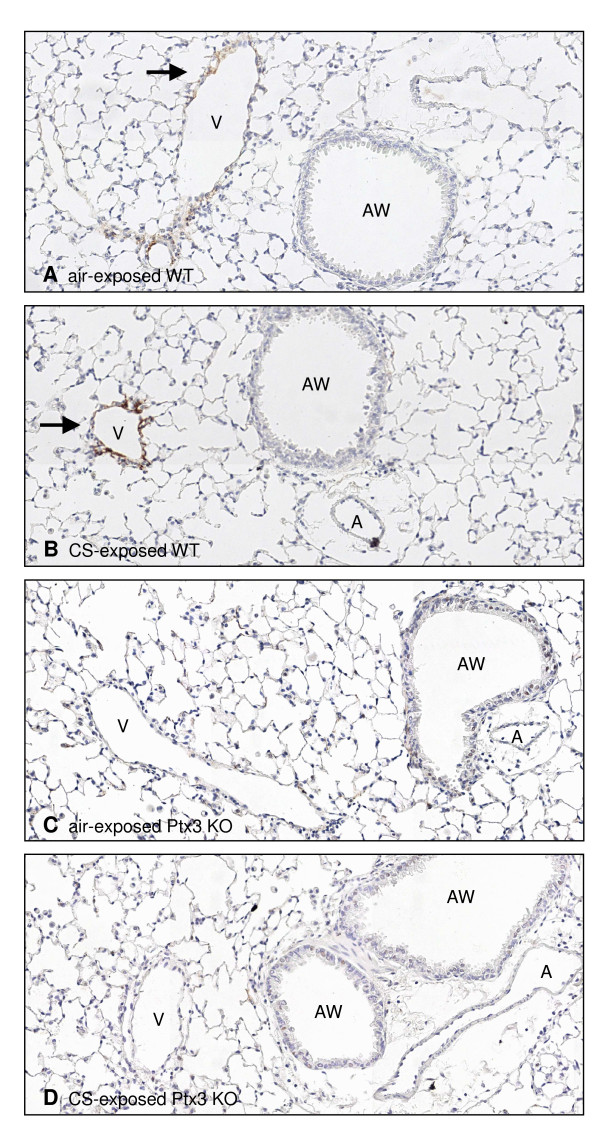
**Pulmonary PTX3 expression by immunohistochemistry (IHC)**. Photomicrographs of PTX3 stained lung tissue of (A) air-exposed WT mice, (B) CS-exposed WT mice, (C) air-exposed Ptx3 KO mice and (D) CS-exposed Ptx3 KO mice. All mice were exposed to air or CS for 4 weeks (subacute). (V: vein; A: artery; AW; airway-magnification 200×).

**Figure 3:**
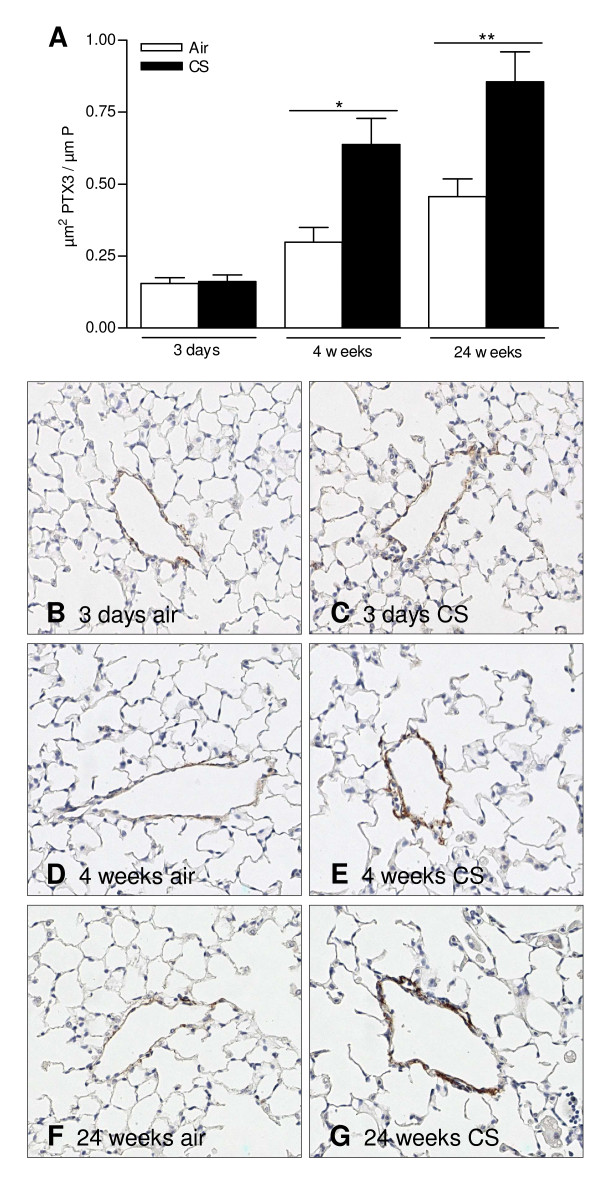
**PTX3 expression in lung veins and venules**. (A) Quantification of PTX3 expression in endothelial cell layer of lung blood vessels. The amount of PTX3 expression measured in the acute (3 days), subacute (4 weeks) or chronic (24 weeks) experiment was normalized for the length of internal perimeter of the vein and expressed as mean ± SEM (N = 8 animals/group; * p < 0.05 and ** p < 0.01). Photomicrographs of PTX3 immunostained veins in lung tissue upon (B) 3 days air, (C) 3 days CS, (D) 4 weeks air, (E) 4 weeks CS, (F) 24 weeks air and (G) 24 weeks CS exposure. (magnification: 200×).

We evaluated pentraxin-3 (PTX3) expression in bronchoalveolar lavage (BAL) fluid and lung tissue of WT mice at 24 h after 3 days (acute), 4 weeks (subacute) or 24 weeks (chronic) air or CS exposure. PTX3 levels were below the detection limit in BAL fluid of both air-and CS-exposed mice, as measured by ELISA (data not shown). In contrast, PTX3 was detectable in lung tissue homogenates at all timepoints and increased significantly upon 4 weeks and 24 weeks of CS exposure (Figure 1). In order to localize pulmonary PTX3, we performed immunohistochemical staining on lung tissue sections using an antibody specific for PTX3. PTX3 was localized in endothelial cells of veins and venules but not in endothelial cells of arteries or other structural lung cells (Figure 2A and 2B). No expression of PTX3 was observed in lungs of Ptx3 KO mice (Figure 2C and 2D). Next, we quantified PTX3 expression in pulmonary veins using the KS400 image analysis platform. In accordance with the ELISA for PTX3 in lung homogenate, acute CS exposure did not affect PTX3 expression in the pulmonary veins, while subacute and chronic CS exposure significantly upregulated PTX3 in veins of the lung by two-fold, compared to air-exposed animals (Figure 3).

### CS-induced pulmonary PTX3 expression depends on the IL-1RI pathway

**Figure 4:**
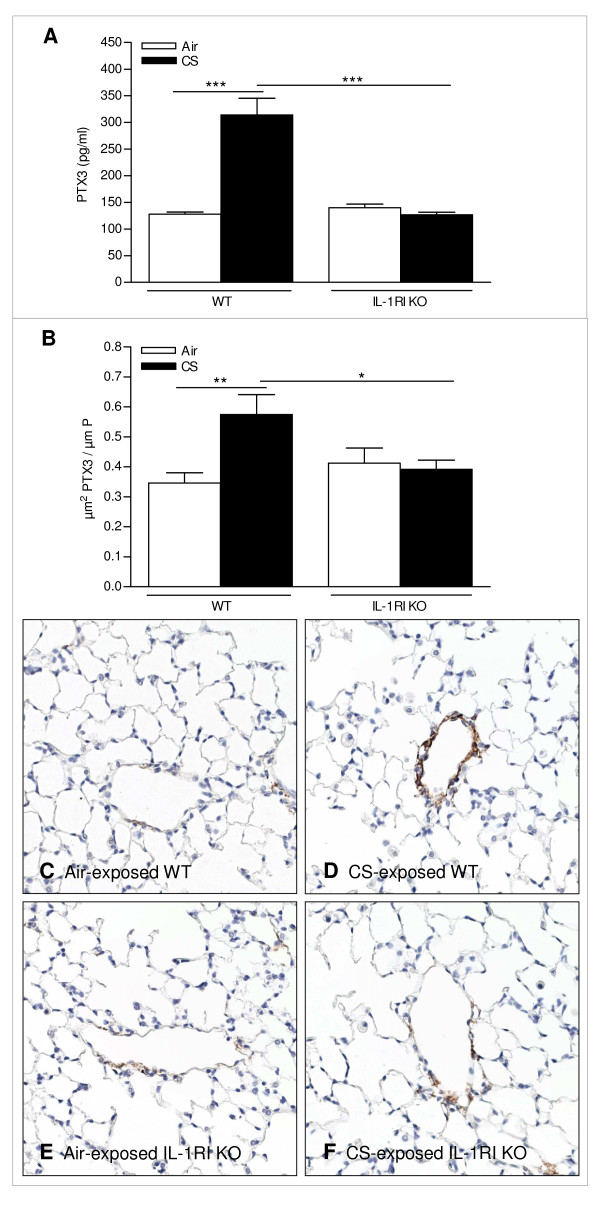
**Effect of IL-1RI deficiency on cigarette smoke (CS)-induced vascular PTX3 expression**. (A) Quantification of PTX3 in lung homogenate by ELISA. (B) Quantification of PTX3 stained endothelial cell layer of lung blood vessels. The amount of PTX3 expression measured in a subacute (4 weeks) experiment was normalized for the length of internal perimeter. Photomicrographs of PTX3 immunostained venules of (C) air-exposed WT, (D) CS-exposed WT, (E) air-exposed IL-1RI KO and (D) CS-exposed IL-1RI KO (magnification: 200×). Data are expressed as mean ± SEM (N = 8-10 animals/group; * p < 0.05, ** p < 0.01 and *** p < 0.01).

Since PTX3 has been identified as an IL-1 inducible gene in endothelial cells [10], we examined PTX3 expression in air-and CS-exposed IL-1RI KO mice. In contrast with the CS-induced increase of PTX3 in WT mice, PTX3 levels in lung homogenates of IL-1RI KO mice were not affected by subacute CS exposure (Figure 4A). Accordingly, CS exposure for 4 weeks did not increase PTX3 expression in veins of IL-1RI KO mice, as analyzed quantitatively on lung sections (Figure 4B). This indicates that the IL-1 pathway regulates CS-induced upregulation of pulmonary vascular PTX3.

### Pulmonary levels of VEGF-A and FGF-2 are affected by CS exposure in a PTX3 independent manner

Since CS exposure affected PTX3 expression in pulmonary veins, we studied growth factors which regulate vascular cell growth and survival, as markers of endothelial function and dysfunction in WT and Ptx3 KO mice. CS significantly downregulated mRNA levels of VEGF-A and FGF-2 at both subacute and chronic timepoints (Table 1). Exposure to CS also significantly affected protein levels of VEGF-A (downregulation) and FGF-2 (upregulation), as documented in Table 1. Interestingly, CS-induced mRNA and protein levels of these growth factors were not affected by PTX3 deficiency (Table 1).

**Table 1:** Expression of VEGF-A and FGF-2 upon subacute and chronic air or CS-exposure in lungs of WT and Ptx3 KO mice

4 weeks				
	**WT-Air**	**WT-CS**	**Ptx3 KO-Air**	**Ptx3 KO-CS**

VEGF-A/HPRT mRNA	1.00 ± 0.04	0.72 ± 0.04**	1.13 ± 0.09	0.78 ± 0.03*

FGF-2/HPRT mRNA	1.03 ± 0.03	0.85 ± 0.04*	1.12 ± 0.05	0.87 ± 0.10*

				

VEGF-A (pg/ml)	610.5 ± 16.8	432.4 ± 18.3***	506.3 ± 16.4	412.5 ± 18.3**

FGF-2 (pg/ml)	2066.3 ± 44.3	3703.8 ± 120.8***	1933.8 ± 101.9	3892.5 ± 96.8***

**24 weeks**				

	**WT-Air**	**WT-CS**	**Ptx3 KO-Air**	**Ptx3 KO-CS**

VEGF-A/HPRT mRNA	1.11 ± 0.06	0.76 ± 0.05**	1.11 ± 0.05	0.65 ± 0.05**

FGF-2/HPRT mRNA	1.12 ± 0.02	0.93 ± 0.02**	1.20 ± 0.09	0.89 ± 0.07*

				

VEGF-A (pg/ml)	527.4 ± 16.0	435.3 ± 17.8**	577.2 ± 10.4	396.00 ± 21.0***

FGF-2 (pg/ml)	1196.1 ± 18.4	1974 ± 62.5***	1163.7 ± 33.0	1946.8 ± 56.0***

Data are presented as mean ± SEM. WT: wild-type, Ptx3 KO: Ptx3 knockout, CS: cigarette smoke.

* p < 0.05, ** p < 0.01 and *** p < 0.001 versus air-exposed mice of same genotype

### CS-induced pulmonary inflammation is not affected in Ptx3 KO mice

**Figure 5:**
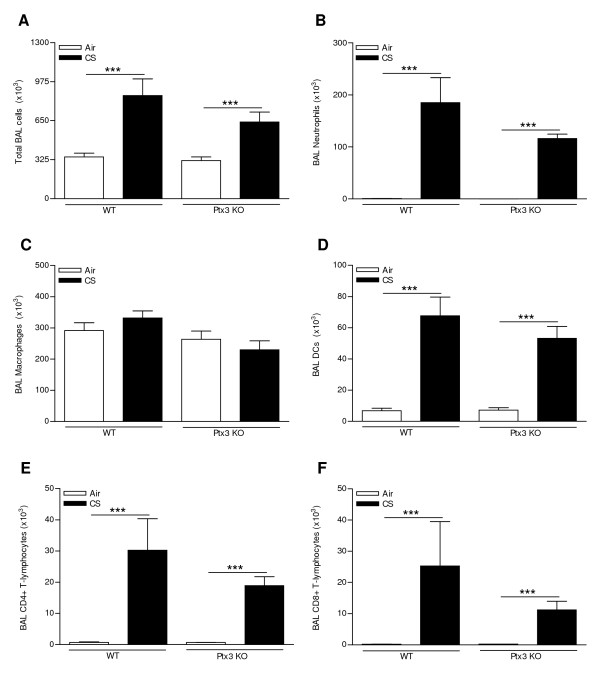
**Effect of subacute (4 weeks) cigarette smoke (CS) exposure and PTX3 deficiency on the cell subsets in bronchoalveolar lavage (BAL) fluid**. (A) Total cell numbers and numbers of (B) neutrophils, (C) macrophages, (D) dendritic cells, (E) CD4+ and (F) CD8+ T-lymphocytes in WT and Ptx3 KO mice upon 4 weeks exposure to air or CS. Results are expressed as mean ± SEM (N = 10 animals/group; *** p < 0.001). All cell types were enumerated by flow cytometry, except for the neutrophils which were determined by cytospin counts.

**Figure 6:**
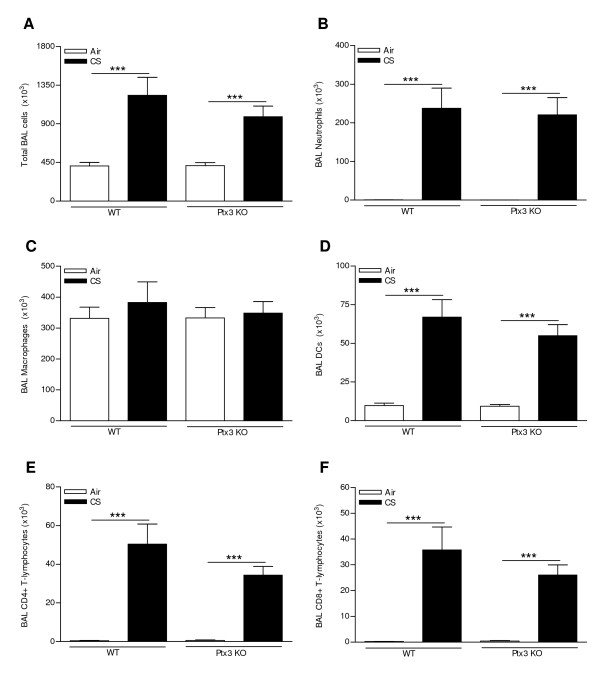
**Effect of chronic (24 weeks) cigarette smoke (CS) exposure and PTX3 deficiency on the cell subsets in bronchoalveolar lavage (BAL) fluid**. (A) Total cell numbers and numbers of (B) neutrophils, (C) macrophages, (D) dendritic cells, (E) CD4+ and (F) CD8+ T-lymphocytes in WT and Ptx3 KO mice upon 24 weeks exposure to air or CS. Results are expressed as mean ± SEM (N = 10 animals/group; *** p < 0.001). All cell types were enumerated by flow cytometry, except for the neutrophils which were determined by cytospin counts.

**Figure 7:**
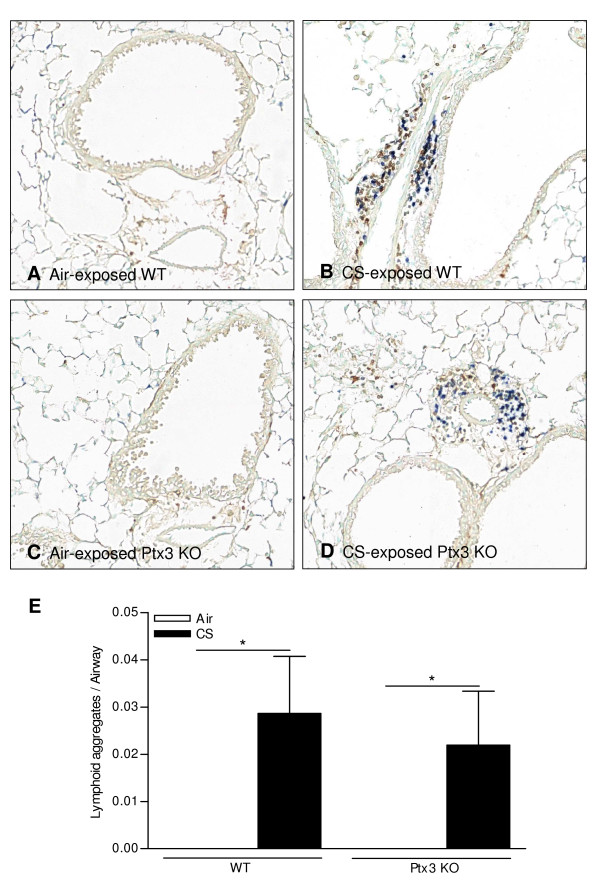
**Effect of cigarette smoke (CS) exposure and PTX3 deficiency on peribronchial lymphoid aggregates formation**. A-D: Photomicrographs of lymphoid aggregates in CD3/B220 immunostained lung tissue of 24 weeks air and CS-exposed WT and Ptx3 KO mice (brown = CD3 positive cells and blue = B220 positive cells). E: Quantification of peribronchial lymphoid aggregates in lung tissue of WT and Ptx3 KO mice upon 24 weeks exposure to air or CS. Data are expressed as mean ± SEM (N = 8 animals/group; * p < 0.05).

PTX3 is a pattern recognition receptor modulating cellular immunity. Therefore, we compared the CS-induced pulmonary inflammation between WT mice and Ptx3 KO mice. Both subacute and chronic CS exposure significantly increased the total numbers of neutrophils, dendritic cells (DCs) and CD4^+ ^and CD8^+ ^T-lymphocytes in BAL fluid of WT and Ptx3 KO mice (Figure 5 and 6). The absolute number of macrophages was not affected by CS exposure or PTX3 deficiency (Figure 5 and 6). However at both timepoints, the CS-induced pulmonary inflammation was not affected by PTX3 deficiency (Figure 5 and 6). Also in lung homogenates, CS-induced accumulation of macrophages, DCs and CD4^+ ^and CD8^+ ^T-lymphocytes was not significantly different between WT and Ptx3 KO mice (data not shown). CS-induced peribronchial lymphoid aggregate formation was also not affected by PTX3 deficiency (Figure 7).

### Emphysema in CS-exposed WT and Ptx3 KO mice

**Figure 8:**
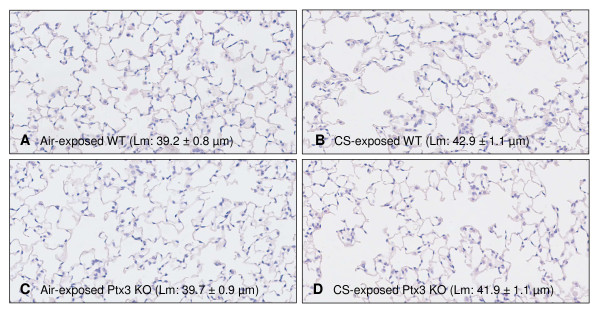
**Effect of cigarette smoke (CS) exposure and PTX3 deficiency on pulmonary emphysema**. (A-D) Photomicrographs of hematoxylin and eosin stained lung tissue of 24 weeks air-and CS-exposed WT and Ptx3 KO mice. Lm (mean linear intercept) results are expressed as mean ± SEM (N = 8 animals/group).

Since PTX3 maintains homeostatic equilibrium in the local immune responses and dysregulation of lung homeostasis can contribute to the pathogenesis of pulmonary emphysema, we examined the mean linear intercept (Lm), a measure of alveolar space enlargement, in WT and Ptx3 KO mice (N = 8 animals/group). In WT mice, Lm increased significantly by 9.4% upon 24 weeks of CS exposure (air: 39.2 ± 0.8 μm vs. CS: 42.9 ± 1.1 μm; p < 0.05; Figure 8A and 8B). In Ptx3 KO mice, Lm increased by 5,5% upon CS exposure (air: 39.7 ± 0.9 μm vs. CS: 41.9 ± 1.1 μm; p > 0.05; Figure 8C and 8D). Importantly, there was no significant difference in airspace enlargement, as measured by Lm, between CS-exposed WT and Ptx3 KO mice (P = 0.54).

### Pulmonary protease/antiprotease imbalance upon CS exposure is unaffected by PTX3 deficiency

Subacute and chronic CS exposure significantly upregulated matrix metalloproteinase-12 (MMP-12) and tissue inhibitor of matrix metalloproteinase (TIMP-1) in lung tissue of WT and Ptx3 KO mice, as measured by qRT-PCR (Table 2). However, CS-induced upregulation was unaffected by the genotype (Table 2). Interestingly, in air-exposed mice, the protease/antiprotease balance was in favor of the antiprotease, whereas the opposite occurred in CS-exposed mice (air-exposed mice: MMP-12/TIMP-1 ratio < 1; CS-exposed mice: MMP-12/TIMP-1 ratio > 1).

**Table 2:** Expression of MMP-12 and TIMP-1 upon subacute and chronic air or CS-exposure in lungs of WT and Ptx3 KO mice

4 weeks				
	**WT-Air**	**WT-CS**	**Ptx3 KO-Air**	**Ptx3 KO-CS**

MMP-12/HPRT mRNA	0.11 ± 0.14	1.91 ± 0.43**	0.09 ± 0.01	1.56 ± 0.17**

TIMP-1/HPRT mRNA	0.44 ± 0.05	1.47 ± 0.32*	0.45 ± 0.03	1.12 ± 0.06**

Ratio MMP-12/TIMP-1	0.25	1.30	0.2	1.39

**24 weeks**				

	**WT-Air**	**WT-CS**	**Ptx3 KO-Air**	**Ptx3 KO-CS**

MMP-12/HPRT mRNA	0.10 ± 0.01	1.66 ± 0.16**	0.10 ± 0.01	1.91 ± 0.43**

TIMP-1/HPRT mRNA	0.72 ± 0.10	1.34 ± 0.10**	0.56 ± 0.05	1.20 ± 0.10**

Ratio MMP-12/TIMP-1	0.14	1.24	0.18	1.60

Data are presented as mean ± SEM. WT: wild-type, Ptx3 KO: Ptx3 knockout, CS: cigarette smoke.

* p < 0.05 and ** p < 0.01 versus air-exposed mice of same genotype

### Rapid but transient upregulation of systemic pentraxin-3 upon CS exposure

**Figure 9:**
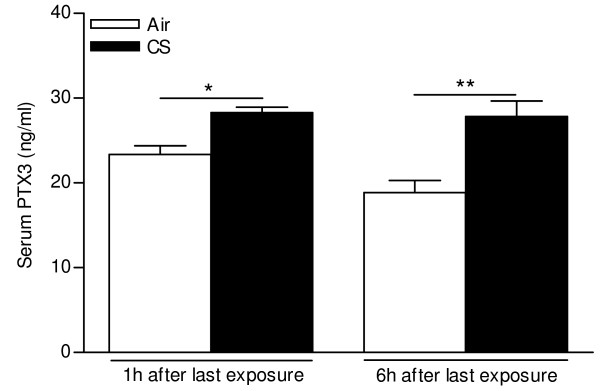
**Effect of acute (3 days) cigarette smoke (CS) exposure on serum PTX3 levels, measured by ELISA at 1 h and 6 h after last exposure**. Data are expressed as mean ± SEM (N = 6 animals/group; * p < 0.05 and ** p < 0.01).

First, we measured serum PTX3 levels of WT mice at 24 h after 3 days (acute), 4 weeks (subacute) or 24 weeks (chronic) air or CS exposure, by ELISA. PTX3 was detectable in serum, but not elevated from baseline levels at 24 h after last smoke exposure at all timepoints (data not shown). Secondly, since PTX3 reacts as an acute phase protein which is rapidly released into the blood stream, we performed an acute (3 days) experiment where mice were sacrificied at earlier timepoints (1 h and 6h) after the last exposure and measured serum PTX3. Indeed, serum PTX3 levels were significantly increased at 1 h and 6 h after the last CS exposure (Figure 9).

### CS exposure induces a failure to gain weight independent of PTX3

In order to evaluate the influence of CS and PTX3 deficiency on body weight, we measured final body weight of mice who were age-and weight-matched at the beginning of the experiment. In accordance with our previous study [29], a significant failure to gain weight was observed in chronically CS-exposed WT mice compared to air-exposed littermates (air-exposed WT mice: 32.33 ± 0.49 g, CS-exposed WT mice: 25.77 ± 0.61 g, P < 0,001). Interestingly, Ptx3 KO mice also failed to gain weight upon chronic CS exposure (air-exposed Ptx3 KO mice: 32.11 ± 0.61 g, CS-exposed Ptx3 KO mice: 27.79 ± 0.58 g, P < 0,001) and there was no statistical significant difference between the two genotypes (p > 0,05).

## Discussion

In this cigarette smoke model of COPD, we demonstrated that subacute and chronic CS exposure significantly upregulates PTX3 expression in endothelial cells of lung veins. Moreover, CS-induced PTX3 upregulation in pulmonary veins is dependent on the IL-1 pathway. However, PTX3 deficiency does not affect several pulmonary hallmarks of COPD such as inflammation, peribronchial lymphoid neogenesis and emphysema. Serum PTX3 levels elevate rapidly but transiently upon CS exposure, but PTX3 deficiency does not influence CS-induced weight changes, a systemic manifestation of COPD.

In this study, we demonstrated that long time CS exposure significantly upregulates pulmonary PTX3 expression in veins in an IL-1 dependent manner. This suggests that PTX3 is more downstream than IL-1 in the inflammatory cascade activated by CS. Recently, Sapey et al. described a correlation of IL-1β with clinical aspects of COPD severity [31]. The contribution of IL-1β in disease pathogenesis was also established using transgenic and KO mouse models of COPD [32-34]. This suggests that IL-1β may play a critical role in COPD, by influencing inflammatory responses. Importantly, IL-1 is also implicated in the induction and maintenance of angiogenesis [35]. Taken together, IL-1 might therefore be involved in tuning vascular inflammatory responses or angiogenesis in smokers and patients with COPD via PTX3. However, further studies of angiogenesis *in vivo *are needed to further address this hypothesis.

Until now, it is unclear whether PTX3 has a protective or a destructive role in (cardio)vascular pathogenesis. On one hand, PTX3 has cardioprotective and atheroprotective roles, as described in mouse models of acute myocardial infarction (AMI) and atherosclerosis [22,36], respectively. On the other hand, a detrimental role was also ascribed to PTX3 in a mouse model of ischemia followed by reperfusion of the superior mesenteric artery [37]. Since exposure to environmental CS induces pulmonary angiogenesis in mice [20], we evaluated VEGF-A and FGF-2-two crucial mediators of angiogenesis and markers of endothelial function-to assign if PTX3 has a protective or destructive role in pulmonary veins of CS-exposed mice. Camozzi et al. described that the characteristic N-terminal domain of PTX3 is able to bind FGF-2, thereby inhibiting the pro-angiogenic activity of FGF-2 [18]. We observed that FGF-2 protein levels significantly increased, whereas FGF-2 mRNA levels decreased, upon 4 weeks and 24 weeks CS exposure. In accordance with our findings, Conte et al. described that FGF-2 is not activated at the transcriptional level but regulated at the translational level in an ischemia model [38]. We observed that both VEGF-A and FGF-2 levels were affected by CS exposure, but the levels were similarly affected in Ptx3 KO and WT mice. Further studies are needed to address if the affected levels of VEGF-A and FGF-2 modulate angiogenesis *in vivo*.

In lungs of patients with COPD, inflammatory responses play an important role in disease pathogenesis [39] and formation of lymphoid follicles [40]. In this murine model, we are able to mimick several hallmarks of COPD pathogenesis [24,29,30]. In this study, CS exposure provoked inflammatory cell accumulation and peribronchial lymphoid aggregate formation, which appeared to be independent of PTX3. Apparently, the role of PTX3 in lung inflammation is stimulus dependent. Garlanda et al. described a massive inflammatory response in lungs of Ptx3 KO mice infected with Aspergillus conidia, compared to WT mice [13]. PTX3 deficiency was also not critical in terms of neutrophilic inflammation in the lungs and cytokine profile in a mouse model of LPS-induced toxicity [13], which is in accordance with our findings. Recently, Deban et al. described an interaction of PTX3-released from cells of bone marrow origin, most likely neutrophils-with P-selectin thereby dampening P-selectin dependent leukocyte recruitment [41]. Our data suggest that in CS-induced inflammation such a negative feedback mechanism is not implicated, possibly because of the chronic character of the CS-induced inflammation and because CS is a mild stimulus for PTX3 induction and release, when compared to LPS or other stimuli used in the study by Deban et al. [41]. Importantly, in a Klebsiella-induced pneumonia model, PTX3 is protective when a low inoculum is given to the mice but detrimental when a high inoculum is given [42]. These different results may reflect varied biological actions of PTX3 depending on the type and intensity of the inflammatory stimulus. Additionaly, the role of PTX3 also depends on the involvement of the different pathogenetic mechanisms, which are known to be modulated by PTX3 (microbial clearance, complement activation, P-selectin-dependent leukocyte recruitment or FGF2-dependent angiogenesis).

Emphysema, a structural disorder of the lung parenchyma, is another hallmark of COPD. In this study, Lm values, a measure of airspace enlargement, were not different between the two CS-exposed genotypes. Inflammation, apoptosis/proliferation imbalance and protease/antiprotease imbalance are the concepts used to explain the pathogenesis of CS-induced emphysema and were investigated in this study [3,43]. As mentioned above, CS-induced inflammatory responses were similar between WT and Ptx3 KO mice. To investigate the apoptosis/proliferation balance, we measured VEGF-A RNA and protein levels in lung tissue. VEGF-A is known to maintain the homeostasis of the alveolar compartment and therefore, decreased VEGF signalling affects the pathogenesis of emphysema as described in human emphysema patients by Kanazawa et al. [44] and animal models of emphysema by Voelkel et al. [43]. VEGF-A levels decreased in CS-exposed mice independently of PTX3. We next examined the protease/antiprotease balance by measuring MMP-12/TIMP-1 ratios. As expected, CS shifted the balance in favor of the protease, MMP-12. However, MMP-12/TIMP-1 ratios were not different between WT and Ptx3 KO mice. Taken together, we conclude that PTX3 deficiency did not affect CS-induced pulmonary manifestations.

Although PTX3 is mostly described to be produced locally by resident cells, several peripheral blood inflammatory cells including neutrophils also release PTX3 [8], which might target more distant organs or tissues besides the lungs. Serum PTX3 behaves as an acute-phase response protein, because its levels are low in normal conditions (about 25 ng/ml in the mouse) and increase moderately to dramatically upon inflammation and infection, depending on the type of stimulus [8]. In our study, systemic PTX3 levels increased significantly but mildly at 1-6 hours after short-time CS exposure. This could suggest that 3 days CS is a mild stimulus for PTX3 release, when compared to LPS [11]. In accordance with the study described by Introna et al. [11], we observed the peak of serum PTX3 shortly after CS exposure and PTX3 levels returned to normal levels at 24 h after exposure. Since CS exposure induces systemic PTX3 expression and failure to gain body weight in mice, we investigated if the body weight changes were mediated by PTX3. Moreover, PTX3 is also expressed in adipose tissue and serum PTX3 levels are higher in genetically obese mice as compared with WT mice [45]. However, the PTX3 deficiency had no effect on the CS-induced failure to gain weight.

Cardiovascular disease contributes significantly to morbidity and mortality in patients with COPD [46]. COPD is associated with chronic systemic inflammation which may contribute to the increased risk of cardiovascular events. C-reactive protein (CRP) is a short pentraxin used as serum biomarker of systemic inflammation in COPD and increased levels of high sensitivity CRP in serum of patients with COPD have been associated with increased mortality [47]. The prototypic long pentraxin PTX3 is also associated with cardiovascular disease independently of CRP [48]. PTX3 is, in contrast with CRP, highly conserved between mice and humans and might therefore be associated with cardiovascular disease in COPD. However in this study, systemic PTX3 levels are only mildly upregulated in CS-exposed mice. Moreover, CS exposure alone is not sufficient to induce atherosclerotic lesions in mice (Pieter Hiemstra, personal communication). Double-knockout mice lacking PTX3 and apolipoprotein E (ApoE) in combination with an atherogenic diet are susceptible for atherosclerosis [36]. In future work, the contribution of CS-exposure to the development of atherosclerosis in Ptx3/ApoE double KO mice has to be elucidated.

## Conclusions

Cigarette smoke induce *in vivo *PTX3 expression in lung endothelial cells of veins in an IL-1 dependent manner. CS exposure also rapidly and transiently enhance systemic PTX3 expression. However, PTX3 deficiency is not critical in CS-induced pulmonary inflammation, peribronchial lympoid neogenesis, emphysema and body weight changes in this murine model of COPD.

## Competing interests

The authors (NSP, KRB, TM, GRVP, CG, AM, GFJ and GGB) declare that they have no competing interests

## Authors' contributions

NSP carried out the design and coordination of the study, performed the data and statistical analysis, and drafted the manuscript. KRB and GGB participated in the design and coordination of the study and helped to interpret the data. All authors (NSP, KRB, TM, GRVP, CG, AM, GFJ and GGB) critically revised the manuscript and approved the final manuscript.

## Authors' Information

KRB is a postdoctoral researcher of the fund for Scientific Research Flanders (FWO Vlaanderen). TM is sponsored by the Interuniversity Attraction Poles Program, Belgian State, Belgian Science Policy, Project P6/35. GRVP is a doctoral researcher of the fund for Scientific Research Flanders (FWO Vlaanderen).

## References

[B1] Pauwels RA Rabe KF Burden and clinical features of chronic obstructive pulmonary disease (COPD) Lancet 2004 364 613 620 10.1016/S0140-6736(04)16855-4 15313363

[B2] Rabe KF Hurd S Anzueto A Barnes PJ Buist SA Calverley P Fukuchi Y Jenkins C Rodriguez-Roisin R van WC Global strategy for the diagnosis, management, and prevention of chronic obstructive pulmonary disease: GOLD executive summary Am J Respir Crit Care Med 2007 176 532 555 10.1164/rccm.200703-456SO 17507545

[B3] Demedts IK Demoor T Bracke KR Joos GF Brusselle GG Role of apoptosis in the pathogenesis of COPD and pulmonary emphysema Respir Res 2006 7 53 10.1186/1465-9921-7-53 16571143 PMC1501017

[B4] Peinado VI Pizarro S Barbera JA Pulmonary vascular involvement in COPD Chest 2008 134 808 814 10.1378/chest.08-0820 18842913

[B5] Garcia-Rio F Miravitlles M Soriano JB Munoz L Duran-Tauleria E Sanchez G Sobradillo V Ancochea J Systemic inflammation in chronic obstructive pulmonary disease: a population-based study Respir Res 2010 11 63 20500811 10.1186/1465-9921-11-63 PMC2891677

[B6] Barnes PJ Celli BR Systemic manifestations and comorbidities of COPD Eur Respir J 2009 33 1165 1185 10.1183/09031936.00128008 19407051

[B7] Agusti A Soriano JB COPD as a systemic disease COPD 2008 5 133 138 10.1080/15412550801941349 18415812

[B8] Bottazzi B Garlanda C Cotena A Moalli F Jaillon S Deban L Mantovani A The long pentraxin PTX3 as a prototypic humoral pattern recognition receptor: interplay with cellular innate immunity Immunol Rev 2009 227 9 18 10.1111/j.1600-065X.2008.00719.x 19120471

[B9] Alles VV Bottazzi B Peri G Golay J Introna M Mantovani A Inducible expression of PTX3, a new member of the pentraxin family, in human mononuclear phagocytes Blood 1994 84 3483 3493 7949102

[B10] Breviario F d'Aniello EM Golay J Peri G Bottazzi B Bairoch A Saccone S Marzella R Predazzi V Rocchi M Interleukin-1-inducible genes in endothelial cells. Cloning of a new gene related to C-reactive protein and serum amyloid P component J Biol Chem 1992 267 22190 22197 1429570

[B11] Introna M Alles VV Castellano M Picardi G De GL Bottazzai B Peri G Breviario F Salmona M De GL Cloning of mouse ptx3, a new member of the pentraxin gene family expressed at extrahepatic sites Blood 1996 87 1862 1872 8634434

[B12] Hasday JD Bascom R Costa JJ Fitzgerald T Dubin W Bacterial endotoxin is an active component of cigarette smoke Chest 1999 115 829 835 10.1378/chest.115.3.829 10084499

[B13] Garlanda C Hirsch E Bozza S Salustri A De Acetis M Nota R Maccagno A Riva F Bottazzi B Peri G Non-redundant role of the long pentraxin PTX3 in anti-fungal innate immune response Nature 2002 420 182 186 10.1038/nature01195 12432394

[B14] Rovere P Peri G Fazzini F Bottazzi B Doni A Bondanza A Zimmermann VS Garlanda C Fascio U Sabbadini MG The long pentraxin PTX3 binds to apoptotic cells and regulates their clearance by antigen-presenting dendritic cells Blood 2000 96 4300 4306 11110705

[B15] Murphy TF Sethi S Klingman KL Brueggemann AB Doern GV Simultaneous respiratory tract colonization by multiple strains of nontypeable haemophilus influenzae in chronic obstructive pulmonary disease: implications for antibiotic therapy J Infect Dis 1999 180 404 409 10.1086/314870 10395856

[B16] Imai K Mercer BA Schulman LL Sonett JR D'Armiento JM Correlation of lung surface area to apoptosis and proliferation in human emphysema Eur Respir J 2005 25 250 258 10.1183/09031936.05.00023704 15684288

[B17] Kasahara Y Tuder RM Cool CD Lynch DA Flores SC Voelkel NF Endothelial cell death and decreased expression of vascular endothelial growth factor and vascular endothelial growth factor receptor 2 in emphysema Am J Respir Crit Care Med 2001 163 737 744 11254533 10.1164/ajrccm.163.3.2002117

[B18] Camozzi M Rusnati M Bugatti A Bottazzi B Mantovani A Bastone A Inforzato A Vincenti S Bracci L Mastroianni D Identification of an antiangiogenic FGF2-binding site in the N terminus of the soluble pattern recognition receptor PTX3 J Biol Chem 2006 281 22605 22613 10.1074/jbc.M601023200 16769728

[B19] Fazzini F Peri G Doni A Dell'Antonio G Dal CE Bozzolo E D'Auria F Praderio L Ciboddo G Sabbadini MG PTX3 in small-vessel vasculitides: an independent indicator of disease activity produced at sites of inflammation Arthritis Rheum 2001 44 2841 2850 10.1002/1529-0131(200112)44:12<2841::AID-ART472>3.0.CO;2-6 11762945

[B20] Rao SP Sikora L Hosseinkhani MR Pinkerton KE Sriramarao P Exposure to environmental tobacco smoke induces angiogenesis and leukocyte trafficking in lung microvessels Exp Lung Res 2009 35 119 135 10.1080/01902140802449729 19263281 PMC3755616

[B21] Peri G Introna M Corradi D Iacuitti G Signorini S Avanzini F Pizzetti F Maggioni AP Moccetti T Metra M PTX3, A prototypical long pentraxin, is an early indicator of acute myocardial infarction in humans Circulation 2000 102 636 641 10931803 10.1161/01.cir.102.6.636

[B22] Salio M Chimenti S De AN Molla F Maina V Nebuloni M Pasqualini F Latini R Garlanda C Mantovani A Cardioprotective function of the long pentraxin PTX3 in acute myocardial infarction Circulation 2008 117 1055 1064 10.1161/CIRCULATIONAHA.107.749234 18268142

[B23] Gan WQ Man SF Senthilselvan A Sin DD Association between chronic obstructive pulmonary disease and systemic inflammation: a systematic review and a meta-analysis Thorax 2004 59 574 580 10.1136/thx.2003.019588 15223864 PMC1747070

[B24] D'hulst AI Vermaelen KY Brusselle GG Joos GF Pauwels RA Time course of cigarette smoke-induced pulmonary inflammation in mice Eur Respir J 2005 26 204 213 10.1183/09031936.05.00095204 16055867

[B25] Bracke KR D'hulst AI Maes T Demedts IK Moerloose KB Kuziel WA Joos GF Brusselle GG Cigarette smoke-induced pulmonary inflammation, but not airway remodeling, is attenuated in chemokine receptor 5-deficient mice Clin Exp All 2007 37 1467 1479 10.1111/j.1365-2222.2007.02808.x 17883726

[B26] Vermaelen KY Carro-Muino I Lambrecht BN Pauwels RA Specific migratory dendritic cells rapidly transport antigen from the airways to the thoracic lymph nodes J Exp Med 2001 193 51 60 10.1084/jem.193.1.51 11136820 PMC2195883

[B27] Vermaelen K Pauwels R Accurate and simple discrimination of mouse pulmonary dendritic cell and macrophage populations by flow cytometry: methodology and new insights Cytometry A 2004 61 170 177 10.1002/cyto.a.20064 15382026

[B28] D'hulst AI Maes T Bracke KR Demedts IK Tournoy KG Joos GF Brusselle GG Cigarette smoke-induced pulmonary emphysema in *scid*-mice. Is the acquired immune system required? Respir Res 2005 6 147 10.1186/1465-9921-6-147 16359546 PMC1334210

[B29] D'hulst AI Bracke KR Maes T De Bleecker JL Pauwels RA Joos GF Brusselle GG Role of tumour necrosis factor-alpha receptor p75 in cigarette smoke-induced pulmonary inflammation and emphysema Eur Respir J 2006 28 102 112 10.1183/09031936.06.00059305 16540505

[B30] Bracke KR D'hulst AI Maes T Moerloose KB Demedts IK Lebecque S Joos GF Brusselle GG Cigarette smoke-induced pulmonary inflammation and emphysema are attenuated in CCR6-deficient mice J Immunol 2006 177 4350 4359 16982869 10.4049/jimmunol.177.7.4350

[B31] Sapey E Ahmad A Bayley D Newbold P Snell N Rugman P Stockley RA Imbalances between interleukin-1 and tumor necrosis factor agonists and antagonists in stable COPD J Clin Immunol 2009 29 508 516 10.1007/s10875-009-9286-8 19291375

[B32] Churg A Zhou S Wang X Wang R Wright JL The role of interleukin-1beta in murine cigarette smoke-induced emphysema and small airway remodeling Am J Respir Cell Mol Biol 2009 40 482 490 10.1165/rcmb.2008-0038OC 18931327

[B33] Lucey EC Keane J Kuang PP Snider GL Goldstein RH Severity of elastase-induced emphysema is decreased in tumor necrosis factor-alpha and interleukin-1beta receptor-deficient mice Lab Invest 2002 82 79 85 11796828 10.1038/labinvest.3780397

[B34] Lappalainen U Whitsett JA Wert SE Tichelaar JW Bry K Interleukin-1beta causes pulmonary inflammation, emphysema, and airway remodeling in the adult murine lung Am J Respir Cell Mol Biol 2005 32 311 318 10.1165/rcmb.2004-0309OC 15668323

[B35] Carmi Y Voronov E Dotan S Lahat N Rahat MA Fogel M Huszar M White MR Dinarello CA Apte RN The role of macrophage-derived IL-1 in induction and maintenance of angiogenesis J Immunol 2009 183 4705 4714 10.4049/jimmunol.0901511 19752225

[B36] Norata GD Marchesi P Pulakazhi V Pasqualini F Anselmo A Moalli F Pizzitola I Garlanda C Mantovani A Catapano AL Deficiency of the long pentraxin PTX3 promotes vascular inflammation and atherosclerosis Circulation 2009 120 699 708 10.1161/CIRCULATIONAHA.108.806547 19667236

[B37] Souza DG Amaral FA Fagundes CT Coelho FM Arantes RM Sousa LP Matzuk MM Garlanda C Mantovani A Dias AA The long pentraxin PTX3 is crucial for tissue inflammation after intestinal ischemia and reperfusion in mice Am J Pathol 2009 174 1309 1318 10.2353/ajpath.2009.080240 19286566 PMC2671362

[B38] Conte C Riant E Toutain C Pujol F Arnal JF Lenfant F Prats AC FGF2 translationally induced by hypoxia is involved in negative and positive feedback loops with HIF-1alpha PLoS One 2008 3 e3078 10.1371/journal.pone.0003078 18728783 PMC2518102

[B39] Cosio MG Saetta M Agusti A Immunologic aspects of chronic obstructive pulmonary disease N Engl J Med 2009 360 2445 2454 10.1056/NEJMra0804752 19494220

[B40] Brusselle GG Demoor T Bracke KR Brandsma CA Timens W Lymphoid follicles in (very) severe COPD: beneficial or harmful? Eur Respir J 2009 34 219 230 10.1183/09031936.00150208 19567605

[B41] Deban L Russo RC Sironi M Moalli F Scanziani M Zambelli V Cuccovillo I Bastone A Gobbi M Valentino S Regulation of leukocyte recruitment by the long pentraxin PTX3 Nat Immunol 2010 11 328 334 10.1038/ni.1854 20208538

[B42] Soares AC Souza DG Pinho V Vieira AT Nicoli JR Cunha FQ Mantovani A Reis LF Dias AA Teixeira MM Dual function of the long pentraxin PTX3 in resistance against pulmonary infection with Klebsiella pneumoniae in transgenic mice Microbes Infect 2006 8 1321 1329 10.1016/j.micinf.2005.12.017 16697676

[B43] Voelkel NF Vandivier RW Tuder RM Vascular endothelial growth factor in the lung Am J Physiol Lung Cell Mol Physiol 2006 290 L209 L221 10.1152/ajplung.00185.2005 16403941

[B44] Kanazawa H Role of vascular endothelial growth factor in the pathogenesis of chronic obstructive pulmonary disease Med Sci Monit 2007 13 RA189 RA195 17968307

[B45] Abderrahim-Ferkoune A Bezy O Chiellini C Maffei M Grimaldi P Bonino F Moustaid-Moussa N Pasqualini F Mantovani A Ailhaud G Characterization of the long pentraxin PTX3 as a TNFalpha-induced secreted protein of adipose cells J Lipid Res 2003 44 994 1000 10.1194/jlr.M200382-JLR200 12611905

[B46] Macnee W Maclay J McAllister D Cardiovascular injury and repair in chronic obstructive pulmonary disease Proc Am Thorac Soc 2008 5 824 833 10.1513/pats.200807-071TH 19017737 PMC2643206

[B47] Man SF Connett JE Anthonisen NR Wise RA Tashkin DP Sin DD C-reactive protein and mortality in mild to moderate chronic obstructive pulmonary disease Thorax 2006 61 849 853 10.1136/thx.2006.059808 16738034 PMC2104755

[B48] Jenny NS Arnold AM Kuller LH Tracy RP Psaty BM Associations of pentraxin 3 with cardiovascular disease and all-cause death: the Cardiovascular Health Study Arterioscler Thromb Vasc Biol 2009 29 594 599 10.1161/ATVBAHA.108.178947 19164811 PMC2661204

